# *catena*-Poly[[penta-μ-benzoato-μ-chlorido-dioxanedineodymium(III)] dioxane 2.5-solvate]

**DOI:** 10.1107/S1600536812017746

**Published:** 2012-04-28

**Authors:** Jeffrey A. Bertke, Allen G. Oliver, Kenneth W. Henderson

**Affiliations:** a 251 Nieuwland Science Hall, Department of Chemistry and Biochemistry, University of Notre Dame, Notre Dame, IN 46556-5670, USA

## Abstract

The asymmetric unit of the title compound, [Nd_2_(C_6_H_5_COO)_5_Cl(C_4_H_8_O_2_)]·2.5C_4_H_8_O_2_, consists of two Nd^III^ ions bridged by one Cl^−^ ion, five benzoate ions and one coordinating 1,4-dioxane mol­ecule. One Nd^III^ ion is nine-coordinate, with a very distorted monocapped square-anti­prismatic geometry. It is coordinated by two chelating carboxyl­ate groups, three monodentate carboxyl­ate groups, one chloride ion and one dioxane mol­ecule. A second independent Nd^III^ ion is eight-coordinated in a distorted square-anti­prismatic geometry by one chelating carboxyl­ate group, five monodentate carboxyl­ate groups and one chloride ion. The chains of the extended structure are parallel to the crystallographic *b* axis. There is a small amount of void space which is filled with five disordered dioxane solvent mol­ecules per unit cell. The intensity contribution of the disordered solvent molecules was removed by applying the *SQUEEZE* procedure in *PLATON* [Spek (2009). *Acta Cryst.* D**65**, 148–155].

## Related literature

For recent research on ditopic-linked secondary building units, see: Morris *et al.* (2008 ▶). For 2-D neodymium adducts, see: Nayak *et al.* (2010 ▶). For the synthesis of the neodymium precursor, see: Andersen *et al.* (1978 ▶). For SQUEEZE analysis of the data, see: Spek (2009 ▶).
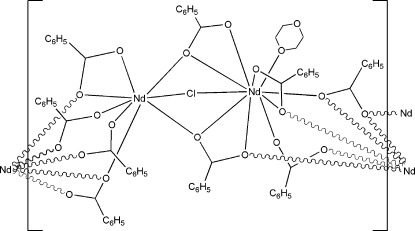


## Experimental

### 

#### Crystal data

[Nd_2_(C_7_H_5_O_2_)_5_Cl(C_4_H_8_O_2_)]·2.5C_4_H_8_O_2_
*M* *_r_* = 1237.84
Triclinic, 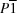

*a* = 12.7631 (5) Å
*b* = 13.6077 (6) Å
*c* = 14.2614 (7) Å
α = 102.785 (3)°
β = 96.943 (3)°
γ = 104.316 (3)°
*V* = 2299.68 (18) Å^3^
*Z* = 2
Mo *K*α radiation
μ = 2.37 mm^−1^
*T* = 100 K
0.32 × 0.18 × 0.16 mm

#### Data collection

Bruker APEXII CCD diffractometer
Absorption correction: multi-scan (*SADABS*; Sheldrick, 2008 ▶) *T*_min_ = 0.606, *T*_max_ = 0.685
27066 measured reflections
8104 independent reflections
4870 reflections with *I* > 2σ(*I*)
*R*_int_ = 0.087

#### Refinement

*R*[*F* ^2^ > 2σ(*F* ^2^)] = 0.052
*wR*(*F* ^2^) = 0.119
*S* = 0.90
8104 reflections
427 parameters
H-atom parameters constrained
Δρ_max_ = 1.45 e Å^−3^
Δρ_min_ = −0.94 e Å^−3^

### 

Data collection: *APEX2* (Bruker, 2008 ▶); cell refinement: *SAINT* (Bruker, 2008 ▶; data reduction: *SAINT*; program(s) used to solve structure: *SHELXS97* (Sheldrick, 2008 ▶); program(s) used to refine structure: *SHELXL97* (Sheldrick, 2008 ▶); molecular graphics: *SHELXTL* (Sheldrick, 2008 ▶); software used to prepare material for publication: *SHELXTL* and *publCIF* (Westrip, 2010 ▶).

## Supplementary Material

Crystal structure: contains datablock(s) I, global. DOI: 10.1107/S1600536812017746/fj2541sup1.cif

Structure factors: contains datablock(s) I. DOI: 10.1107/S1600536812017746/fj2541Isup3.hkl

Supplementary material file. DOI: 10.1107/S1600536812017746/fj2541Isup4.cdx

Additional supplementary materials:  crystallographic information; 3D view; checkCIF report

## Figures and Tables

**Fig. 1.**
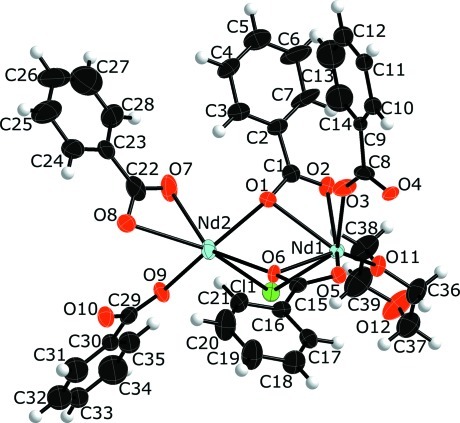
Labelling scheme for I. Thermal ellipsoids represented at 50% probability. H atoms depicted as spheres of a fixed radius.

**Fig. 2.**
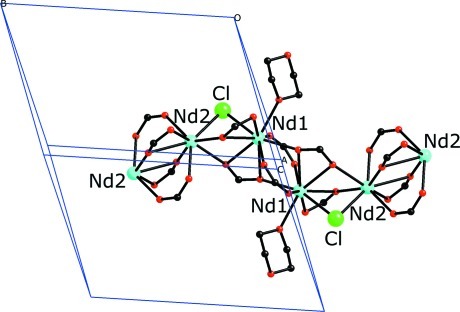
Extended structure, with the one-dimensional chain extending along the b-axis. All phenyl rings of the benzoate ligands and all hydrogen atoms have been removed for clarity.

## supplementary crystallographic information

### Comment

We are interested in building three-dimensional materials using pre-assembled molecular aggregates as secondary building units (SBUs) linked through ditopic organic molecules (Morris *et al.*, 2008). The title compound was synthesized with this goal in mind. However, instead of forming neodymium-benzoate SBUs which could be linked through 1,4-dioxane, a 1-D neodymium-benzoate polymer was crystallized. The closely related compound [Nd_2_(C_6_H_5_COO)_6_(CH_3_OH)_4_] was previously reported as a 2-D material from PXRD data(Nayak *et al.*, 2010).

The asymmetric unit consists of two Nd^3+^ metal centers bridged by one Cl^-^ ion, five benzoate ions and one coordinated 1,4-dioxane molecule (Figure 1). The presence of chloride can be explained as carry-over from the synthesis of Nd(O^i^Pr)_3_ which is synthesized from NdCl_3_ and NaO^i^Pr (Andersen *et al.*, 1978). The extended crystal structure is a 1-D polymeric chain composed of Nd/Cl/carboxylate interactions. The dioxane solvent molecule acts as a terminal donor. The 1-D chains extend along the crystallographic *b*-axis (Figure 2). Small channels also run along the crystallographic *b*-axis and are filled with disordered solvent molecules. The solvent could not be reliably modeled and was omitted through use of the SQUEEZE routine within *PLATON* (Spek, 2009; see refinement procedures for details).

Nd1 adopts a 9-coordinate distorted mono-capped square anti-prismatic geometry. It is coordinated by two chelating carboxylate groups, three monodentate carboxylate groups, one bridging chloride ion and one terminally coordinating dioxane molecule. Nd2 is 8-coordinate in a distorted square anti-prismatic geometry. It is coordinated by one chelating carboxylate group, five monodentate carboxylate groups and the bridging chloride ion.

### Experimental

Benzoic acid was purchased from Aldrich and used without further purification. Nd(OiPr)_3_ was purchased from Strem and used without further purification. 1,4-dioxane was distilled onto molecular sieves from potassium benzophenone.

Benzoic acid (0.366 g, 3 mmol) was dissolved in 1,4-dioxane (20 ml). Solid Nd(O^i^Pr)_3_ (0.321 g, 1 mmol) was added and the solution was refluxed overnight. The solution was allowed to cool to room temperature and then filtered. The filtrate was concentrated and single crystals were grown upon cooling of this saturated solution to room temperature in a hot water bath over the course of several days. Crystalline yield 0.069 g (11.14%) calculated based on Nd.

### Refinement

Hydrogen atoms were included in idealized geometries riding on the atom to which they are bonded. Aromatic C–H distances were constrained to 0.95 Å and methylene C–H distances constrained to 0.99 Å. All hydrogen thermal parameters were set to 1.2 × *U*_eq_ of the carbon to which they are bonded.

Disordered solvent could not be reliably modeled and was thus omitted from the model through the use of SQUEEZE (Spek, 2009). The void space analysis yielded a volume of 540 Å^3^ with an electron count of 224, located at a center of symmetry. This corresponds well to five molecules of dioxane per unit cell. The atom count has been corrected to reflect this solvent inclusion.

### Crystal data

**Table:**

[Nd_2_(C_7_H_5_O_2_)_5_Cl(C_4_H_8_O_2_)]·2.5C_4_H_8_O_2_	*Z* = 2
*M**_r_* = 1237.84	*F*(000) = 1240
Triclinic, *P*1	*D*_x_ = 1.788 Mg m^−^^3^
Hall symbol: -P 1	Mo *K*α radiation, λ = 0.71073 Å
*a* = 12.7631 (5) Å	Cell parameters from 2312 reflections
*b* = 13.6077 (6) Å	θ = 2.4–19.0°
*c* = 14.2614 (7) Å	µ = 2.37 mm^−^^1^
α = 102.785 (3)°	*T* = 100 K
β = 96.943 (3)°	Needle, blue
γ = 104.316 (3)°	0.32 × 0.18 × 0.16 mm
*V* = 2299.68 (18) Å^3^	

### Data collection

**Table:**

Bruker APEXII CCD diffractometer	8104 independent reflections
Radiation source: fine-focus sealed tube	4870 reflections with *I* > 2σ(*I*)
Graphite monochromator	*R*_int_ = 0.087
Detector resolution: 8.33 pixels mm^-1^	θ_max_ = 25.1°, θ_min_ = 1.5°
φ and ω scans	*h* = −15→15
Absorption correction: multi-scan (*SADABS*; Sheldrick, 2008)	*k* = −16→16
*T*_min_ = 0.606, *T*_max_ = 0.685	*l* = −13→17
27066 measured reflections	

### Refinement

**Table:**

Refinement on *F*^2^	Primary atom site location: structure-invariant direct methods
Least-squares matrix: full	Secondary atom site location: difference Fourier map
*R*[*F*^2^ > 2σ(*F*^2^)] = 0.052	Hydrogen site location: inferred from neighbouring sites
*wR*(*F*^2^) = 0.119	H-atom parameters constrained
*S* = 0.90	*w* = 1/[σ^2^(*F*_o_^2^) + (0.0572*P*)^2^] where *P* = (*F*_o_^2^ + 2*F*_c_^2^)/3
8104 reflections	(Δ/σ)_max_ = 0.001
427 parameters	Δρ_max_ = 1.45 e Å^−^^3^
0 restraints	Δρ_min_ = −0.94 e Å^−^^3^

### Special details

**Table:**

Geometry. All e.s.d.'s (except the e.s.d. in the dihedral angle between two l.s. planes) are estimated using the full covariance matrix. The cell e.s.d.'s are taken into account individually in the estimation of e.s.d.'s in distances, angles and torsion angles; correlations between e.s.d.'s in cell parameters are only used when they are defined by crystal symmetry. An approximate (isotropic) treatment of cell e.s.d.'s is used for estimating e.s.d.'s involving l.s. planes.
Refinement. Refinement of *F*^2^ against ALL reflections. The weighted *R*-factor *wR* and goodness of fit *S* are based on *F*^2^, conventional *R*-factors *R* are based on *F*, with *F* set to zero for negative *F*^2^. The threshold expression of *F*^2^ > σ(*F*^2^) is used only for calculating *R*-factors(gt) *etc*. and is not relevant to the choice of reflections for refinement. *R*-factors based on *F*^2^ are statistically about twice as large as those based on *F*, and *R*- factors based on ALL data will be even larger.

### Fractional atomic coordinates and isotropic or equivalent isotropic displacement parameters (Å^2^)

**Table:**

	*x*	*y*	*z*	*U*_iso_*/*U*_eq_	Occ. (<1)
Nd1	0.42843 (3)	0.05580 (3)	0.39858 (3)	0.02441 (14)	
Nd2	0.46041 (4)	0.35430 (3)	0.41815 (4)	0.03265 (16)	
Cl1	0.27807 (15)	0.17311 (16)	0.36816 (16)	0.0329 (5)	
O1	0.5172 (4)	0.2130 (4)	0.3257 (4)	0.0310 (14)	
O2	0.4585 (4)	0.0526 (4)	0.2303 (4)	0.0341 (14)	
O3	0.6189 (4)	0.0707 (5)	0.3970 (5)	0.0456 (17)	
O4	0.7013 (4)	0.0001 (4)	0.5011 (4)	0.0316 (14)	
O5	0.4679 (4)	−0.1024 (4)	0.4236 (4)	0.0300 (13)	
O6	0.5077 (4)	−0.2327 (4)	0.4716 (4)	0.0291 (13)	
O7	0.5231 (7)	0.4308 (5)	0.2902 (5)	0.063 (2)	
O8	0.5524 (4)	0.5571 (4)	0.4251 (4)	0.0368 (15)	
O9	0.3103 (4)	0.4307 (4)	0.3939 (4)	0.0358 (15)	
O10	0.3508 (4)	0.5843 (4)	0.5070 (4)	0.0377 (15)	
O11	0.2669 (4)	−0.0860 (4)	0.2839 (4)	0.0374 (15)	
O12	0.0494 (6)	−0.1857 (6)	0.1846 (5)	0.074 (2)	
C1	0.5171 (7)	0.1464 (7)	0.2475 (6)	0.032 (2)	
C2	0.5931 (7)	0.1778 (7)	0.1806 (6)	0.034 (2)	
C3	0.6589 (7)	0.2799 (7)	0.1979 (7)	0.041 (2)	
H3	0.6534	0.3332	0.2512	0.049*	
C4	0.7321 (7)	0.3036 (8)	0.1375 (7)	0.044 (2)	
H4	0.7758	0.3739	0.1480	0.052*	
C5	0.7424 (9)	0.2266 (9)	0.0625 (8)	0.061 (3)	
H5	0.7949	0.2431	0.0224	0.074*	
C6	0.6765 (11)	0.1250 (10)	0.0450 (9)	0.087 (4)	
H6	0.6828	0.0715	−0.0077	0.104*	
C7	0.6023 (9)	0.1019 (8)	0.1038 (7)	0.062 (3)	
H7	0.5563	0.0321	0.0913	0.074*	
C8	0.7002 (7)	0.0470 (7)	0.4349 (7)	0.036 (2)	
C15	0.4761 (6)	−0.1963 (6)	0.4033 (6)	0.0265 (19)	
C16	0.4459 (6)	−0.2587 (7)	0.2995 (6)	0.029 (2)	
C17	0.4655 (7)	−0.2070 (7)	0.2282 (6)	0.036 (2)	
H17	0.4979	−0.1331	0.2458	0.043*	
C18	0.4375 (8)	−0.2634 (8)	0.1285 (7)	0.049 (3)	
H18	0.4518	−0.2286	0.0786	0.059*	
C19	0.3889 (10)	−0.3704 (8)	0.1055 (8)	0.066 (3)	
H19	0.3696	−0.4097	0.0389	0.079*	
C20	0.3682 (9)	−0.4208 (8)	0.1774 (7)	0.063 (3)	
H20	0.3328	−0.4941	0.1598	0.075*	
C21	0.3980 (6)	−0.3665 (6)	0.2748 (6)	0.033 (2)	
H21	0.3859	−0.4023	0.3243	0.039*	
C22	0.5731 (9)	0.5236 (9)	0.3398 (8)	0.052 (3)	
C23	0.6533 (9)	0.5968 (8)	0.3005 (8)	0.050 (3)	
C24	0.7152 (8)	0.6935 (8)	0.3578 (8)	0.053 (3)	
H24	0.7077	0.7161	0.4240	0.063*	
C25	0.7892 (9)	0.7580 (11)	0.3175 (11)	0.080 (4)	
H25	0.8355	0.8241	0.3564	0.096*	
C26	0.7940 (11)	0.7236 (13)	0.2186 (12)	0.091 (5)	
H26	0.8449	0.7665	0.1905	0.110*	
C27	0.7268 (14)	0.6292 (14)	0.1617 (11)	0.099 (5)	
H27	0.7296	0.6084	0.0941	0.119*	
C28	0.6564 (11)	0.5655 (10)	0.2008 (9)	0.076 (4)	
H28	0.6096	0.5000	0.1612	0.092*	
C29	0.2858 (7)	0.5090 (7)	0.4387 (7)	0.034 (2)	
C36	0.2039 (7)	−0.1803 (7)	0.3002 (8)	0.056 (3)	
H36A	0.2273	−0.1824	0.3683	0.067*	
H36B	0.2163	−0.2415	0.2555	0.067*	
C37	0.0851 (8)	−0.1856 (8)	0.2826 (8)	0.060 (3)	
H37A	0.0415	−0.2503	0.2961	0.072*	
H37B	0.0729	−0.1245	0.3277	0.072*	
C38	0.2271 (8)	−0.0800 (8)	0.1877 (7)	0.060 (3)	
H38A	0.2405	−0.1372	0.1386	0.072*	
H38B	0.2679	−0.0122	0.1778	0.072*	
C39	0.1106 (9)	−0.0887 (10)	0.1734 (9)	0.078 (4)	
H39A	0.0973	−0.0306	0.2216	0.093*	
H39B	0.0858	−0.0825	0.1071	0.093*	
C9	0.8010 (9)	0.0640 (11)	0.3862 (10)	0.021 (4)*	0.50
C10	0.9017 (11)	0.0620 (12)	0.4340 (9)	0.032 (9)*	0.50
H10	0.9062	0.0414	0.4935	0.038*	0.50
C11	0.9959 (8)	0.0900 (11)	0.3948 (10)	0.031 (6)*	0.50
H11	1.0647	0.0886	0.4275	0.037*	0.50
C12	0.9893 (9)	0.1201 (10)	0.3077 (10)	0.041 (5)*	0.50
H12	1.0537	0.1392	0.2810	0.049*	0.50
C13	0.8886 (11)	0.1221 (12)	0.2599 (8)	0.084 (8)*	0.50
H13	0.8842	0.1426	0.2004	0.101*	0.50
C14	0.7945 (9)	0.0941 (12)	0.2991 (10)	0.076 (7)*	0.50
H14	0.7257	0.0955	0.2664	0.092*	0.50
C9A	0.8092 (8)	0.0957 (9)	0.4084 (8)	0.019 (4)*	0.50
C10A	0.9047 (11)	0.0748 (11)	0.4459 (9)	0.040 (10)*	0.50
H10A	0.9030	0.0344	0.4925	0.048*	0.50
C11A	1.0026 (8)	0.1131 (11)	0.4154 (10)	0.032 (6)*	0.50
H11A	1.0678	0.0988	0.4411	0.039*	0.50
C12A	1.0050 (7)	0.1723 (9)	0.3474 (9)	0.037 (4)*	0.50
H12A	1.0720	0.1984	0.3265	0.044*	0.50
C13A	0.9096 (8)	0.1931 (8)	0.3098 (8)	0.042 (5)*	0.50
H13A	0.9113	0.2336	0.2633	0.051*	0.50
C14A	0.8117 (7)	0.1548 (8)	0.3403 (8)	0.022 (3)*	0.50
H14A	0.7465	0.1691	0.3147	0.026*	0.50
C30	0.1778 (10)	0.5269 (13)	0.4109 (13)	0.029 (7)*	0.50
C31	0.1526 (13)	0.6120 (13)	0.4677 (12)	0.066 (10)*	0.50
H31	0.2060	0.6599	0.5210	0.079*	0.50
C32	0.0493 (14)	0.6268 (12)	0.4467 (12)	0.062 (8)*	0.50
H32	0.0321	0.6850	0.4856	0.074*	0.50
C33	−0.0288 (10)	0.5566 (13)	0.3688 (11)	0.043 (5)*	0.50
H33	−0.0994	0.5668	0.3544	0.052*	0.50
C34	−0.0036 (11)	0.4716 (11)	0.3119 (10)	0.089 (8)*	0.50
H34	−0.0570	0.4236	0.2587	0.106*	0.50
C35	0.0997 (12)	0.4567 (10)	0.3329 (10)	0.048 (5)*	0.50
H35	0.1169	0.3986	0.2941	0.058*	0.50
C30A	0.1673 (10)	0.5126 (12)	0.4180 (12)	0.031 (7)*	0.50
C31A	0.1370 (11)	0.6039 (10)	0.4499 (13)	0.032 (6)*	0.50
H31A	0.1903	0.6658	0.4894	0.039*	0.50
C32A	0.0287 (12)	0.6048 (11)	0.4242 (13)	0.035 (5)*	0.50
H32A	0.0080	0.6673	0.4460	0.043*	0.50
C33A	−0.0493 (9)	0.5143 (14)	0.3665 (12)	0.079 (9)*	0.50
H33A	−0.1233	0.5149	0.3489	0.095*	0.50
C34A	−0.0190 (12)	0.4229 (11)	0.3346 (11)	0.098 (9)*	0.50
H34A	−0.0723	0.3611	0.2951	0.117*	0.50
C35A	0.0892 (13)	0.4221 (10)	0.3603 (11)	0.055 (6)*	0.50
H35A	0.1099	0.3596	0.3385	0.066*	0.50

### Atomic displacement parameters (Å^2^)

**Table:**

	*U*^11^	*U*^22^	*U*^33^	*U*^12^	*U*^13^	*U*^23^
Nd1	0.0195 (2)	0.0248 (3)	0.0326 (3)	0.00797 (19)	0.0064 (2)	0.0122 (2)
Nd2	0.0263 (3)	0.0245 (3)	0.0492 (4)	0.0104 (2)	0.0034 (2)	0.0121 (2)
Cl1	0.0200 (10)	0.0315 (11)	0.0472 (14)	0.0080 (8)	0.0016 (9)	0.0118 (10)
O1	0.032 (3)	0.030 (3)	0.033 (4)	0.010 (3)	0.011 (3)	0.008 (3)
O2	0.034 (3)	0.027 (3)	0.039 (4)	0.003 (3)	0.004 (3)	0.011 (3)
O3	0.020 (3)	0.069 (4)	0.064 (4)	0.019 (3)	0.015 (3)	0.040 (4)
O4	0.025 (3)	0.042 (3)	0.044 (4)	0.015 (3)	0.017 (3)	0.031 (3)
O5	0.031 (3)	0.027 (3)	0.036 (4)	0.012 (2)	0.008 (3)	0.011 (3)
O6	0.030 (3)	0.028 (3)	0.037 (4)	0.012 (2)	0.007 (3)	0.018 (3)
O7	0.118 (7)	0.041 (4)	0.048 (5)	0.040 (4)	0.026 (4)	0.021 (4)
O8	0.033 (3)	0.040 (4)	0.044 (4)	0.016 (3)	0.009 (3)	0.015 (3)
O9	0.028 (3)	0.027 (3)	0.051 (4)	0.013 (3)	0.004 (3)	0.005 (3)
O10	0.027 (3)	0.029 (3)	0.055 (4)	0.009 (3)	0.002 (3)	0.009 (3)
O11	0.038 (3)	0.028 (3)	0.044 (4)	0.003 (3)	0.004 (3)	0.014 (3)
O12	0.048 (4)	0.090 (6)	0.059 (5)	−0.025 (4)	−0.019 (4)	0.034 (4)
C1	0.027 (5)	0.038 (5)	0.036 (6)	0.017 (4)	0.003 (4)	0.017 (5)
C2	0.032 (5)	0.048 (6)	0.028 (5)	0.013 (4)	0.008 (4)	0.022 (5)
C3	0.034 (5)	0.052 (6)	0.039 (6)	0.009 (4)	0.008 (4)	0.017 (5)
C4	0.031 (5)	0.048 (6)	0.049 (7)	−0.002 (4)	0.004 (5)	0.024 (5)
C5	0.067 (8)	0.079 (8)	0.043 (7)	0.016 (6)	0.021 (6)	0.027 (6)
C6	0.112 (11)	0.074 (9)	0.062 (8)	−0.009 (8)	0.059 (8)	0.010 (7)
C7	0.072 (8)	0.057 (7)	0.042 (7)	−0.017 (6)	0.027 (6)	0.012 (6)
C8	0.023 (5)	0.044 (5)	0.044 (6)	0.012 (4)	0.014 (4)	0.010 (5)
C15	0.012 (4)	0.032 (5)	0.034 (5)	0.002 (3)	0.002 (4)	0.011 (4)
C16	0.023 (4)	0.043 (5)	0.027 (5)	0.009 (4)	0.001 (4)	0.022 (4)
C17	0.034 (5)	0.035 (5)	0.039 (6)	0.015 (4)	0.005 (4)	0.007 (5)
C18	0.064 (7)	0.055 (7)	0.029 (6)	0.016 (5)	0.003 (5)	0.016 (5)
C19	0.101 (9)	0.046 (7)	0.045 (7)	0.016 (6)	0.000 (6)	0.012 (6)
C20	0.090 (9)	0.042 (6)	0.043 (7)	0.014 (6)	−0.013 (6)	0.003 (6)
C21	0.034 (5)	0.038 (5)	0.036 (6)	0.016 (4)	0.001 (4)	0.024 (4)
C22	0.076 (8)	0.059 (7)	0.044 (7)	0.050 (6)	0.010 (6)	0.029 (6)
C23	0.068 (7)	0.047 (6)	0.062 (8)	0.041 (6)	0.036 (6)	0.031 (6)
C24	0.046 (6)	0.065 (7)	0.063 (7)	0.027 (6)	0.023 (6)	0.029 (6)
C25	0.039 (7)	0.100 (10)	0.115 (12)	0.012 (6)	0.025 (7)	0.058 (9)
C26	0.071 (9)	0.129 (14)	0.108 (13)	0.031 (9)	0.054 (9)	0.076 (11)
C27	0.119 (14)	0.124 (14)	0.081 (11)	0.062 (12)	0.031 (10)	0.042 (11)
C28	0.127 (12)	0.064 (8)	0.063 (9)	0.040 (8)	0.053 (8)	0.036 (7)
C29	0.027 (5)	0.036 (5)	0.049 (6)	0.013 (4)	0.010 (4)	0.023 (5)
C36	0.043 (6)	0.041 (6)	0.081 (8)	0.002 (5)	0.002 (5)	0.026 (6)
C37	0.050 (7)	0.054 (7)	0.073 (8)	0.004 (5)	0.005 (6)	0.024 (6)
C38	0.058 (7)	0.069 (7)	0.045 (7)	−0.002 (6)	−0.010 (5)	0.031 (6)
C39	0.056 (7)	0.083 (9)	0.085 (9)	−0.003 (6)	−0.008 (6)	0.040 (7)

### Geometric parameters (Å, º)

**Table:**

Nd1—O3	2.392 (5)	C22—C23	1.495 (14)
Nd1—O4^i^	2.409 (5)	C23—C24	1.370 (13)
Nd1—O5	2.418 (5)	C23—C28	1.399 (14)
Nd1—O2	2.469 (5)	C24—C25	1.392 (14)
Nd1—O11	2.543 (5)	C24—H24	0.9500
Nd1—O6^i^	2.565 (5)	C25—C26	1.398 (17)
Nd1—O5^i^	2.578 (5)	C25—H25	0.9500
Nd1—O1	2.647 (5)	C26—C27	1.368 (18)
Nd1—Cl1	2.835 (2)	C26—H26	0.9500
Nd1—C1	2.918 (9)	C27—C28	1.349 (17)
Nd1—C15^i^	2.958 (8)	C27—H27	0.9500
Nd1—Nd2	3.9235 (6)	C28—H28	0.9500
Nd2—O8^ii^	2.339 (6)	C29—C30	1.482 (13)
Nd2—O10^ii^	2.373 (5)	C29—C30A	1.521 (13)
Nd2—O1	2.400 (5)	C36—C37	1.488 (13)
Nd2—O7	2.415 (7)	C36—H36A	0.9900
Nd2—O9	2.420 (5)	C36—H36B	0.9900
Nd2—O6^i^	2.589 (5)	C37—H37A	0.9900
Nd2—O8	2.693 (6)	C37—H37B	0.9900
Nd2—Cl1	2.826 (2)	C38—C39	1.447 (14)
Nd2—C22	2.918 (11)	C38—H38A	0.9900
Nd2—Nd2^ii^	3.9428 (9)	C38—H38B	0.9900
O1—C1	1.272 (9)	C39—H39A	0.9900
O2—C1	1.262 (9)	C39—H39B	0.9900
O3—C8	1.258 (10)	C9—C10	1.3900
O4—C8	1.251 (10)	C9—C14	1.3900
O4—Nd1^i^	2.409 (5)	C10—C11	1.3900
O5—C15	1.280 (9)	C10—H10	0.9500
O5—Nd1^i^	2.578 (5)	C11—C12	1.3900
O6—C15	1.254 (9)	C11—H11	0.9500
O6—Nd1^i^	2.565 (5)	C12—C13	1.3900
O6—Nd2^i^	2.589 (5)	C12—H12	0.9500
O7—C22	1.265 (12)	C13—C14	1.3900
O8—C22	1.284 (11)	C13—H13	0.9500
O8—Nd2^ii^	2.339 (6)	C14—H14	0.9500
O9—C29	1.246 (9)	C9A—C10A	1.3900
O10—C29	1.276 (10)	C9A—C14A	1.3900
O10—Nd2^ii^	2.373 (5)	C10A—C11A	1.3900
O11—C36	1.419 (10)	C10A—H10A	0.9500
O11—C38	1.431 (10)	C11A—C12A	1.3900
O12—C39	1.411 (12)	C11A—H11A	0.9500
O12—C37	1.417 (11)	C12A—C13A	1.3900
C1—C2	1.495 (11)	C12A—H12A	0.9500
C2—C7	1.368 (12)	C13A—C14A	1.3900
C2—C3	1.384 (12)	C13A—H13A	0.9500
C3—C4	1.374 (12)	C14A—H14A	0.9500
C3—H3	0.9500	C30—C31	1.3900
C4—C5	1.365 (13)	C30—C35	1.3900
C4—H4	0.9500	C31—C32	1.3900
C5—C6	1.379 (15)	C31—H31	0.9500
C5—H5	0.9500	C32—C33	1.3900
C6—C7	1.366 (13)	C32—H32	0.9500
C6—H6	0.9500	C33—C34	1.3900
C7—H7	0.9500	C33—H33	0.9500
C8—C9A	1.517 (12)	C34—C35	1.3900
C8—C9	1.528 (12)	C34—H34	0.9500
C15—C16	1.486 (11)	C35—H35	0.9500
C15—Nd1^i^	2.958 (8)	C30A—C31A	1.3900
C16—C17	1.373 (11)	C30A—C35A	1.3900
C16—C21	1.387 (11)	C31A—C32A	1.3900
C17—C18	1.413 (12)	C31A—H31A	0.9500
C17—H17	0.9500	C32A—C33A	1.3900
C18—C19	1.380 (13)	C32A—H32A	0.9500
C18—H18	0.9500	C33A—C34A	1.3900
C19—C20	1.370 (14)	C33A—H33A	0.9500
C19—H19	0.9500	C34A—C35A	1.3900
C20—C21	1.378 (12)	C34A—H34A	0.9500
C20—H20	0.9500	C35A—H35A	0.9500
C21—H21	0.9500		
			
O3—Nd1—O4^i^	136.30 (19)	C7—C6—C5	119.5 (11)
O3—Nd1—O5	70.64 (19)	C7—C6—H6	120.2
O4^i^—Nd1—O5	77.57 (18)	C5—C6—H6	120.2
O3—Nd1—O2	72.27 (19)	C6—C7—C2	120.9 (10)
O4^i^—Nd1—O2	145.82 (18)	C6—C7—H7	119.5
O5—Nd1—O2	103.26 (18)	C2—C7—H7	119.5
O3—Nd1—O11	125.9 (2)	O4—C8—O3	125.8 (8)
O4^i^—Nd1—O11	73.60 (18)	O4—C8—C9A	118.0 (8)
O5—Nd1—O11	78.40 (17)	O3—C8—C9A	115.4 (9)
O2—Nd1—O11	73.18 (18)	O4—C8—C9	117.3 (8)
O3—Nd1—O6^i^	86.92 (19)	O3—C8—C9	116.4 (9)
O4^i^—Nd1—O6^i^	86.44 (18)	C9A—C8—C9	17.2 (7)
O5—Nd1—O6^i^	123.61 (17)	O6—C15—O5	119.0 (7)
O2—Nd1—O6^i^	118.35 (17)	O6—C15—C16	122.6 (7)
O11—Nd1—O6^i^	146.72 (17)	O5—C15—C16	118.4 (7)
O3—Nd1—O5^i^	71.19 (19)	O6—C15—Nd1^i^	59.7 (4)
O4^i^—Nd1—O5^i^	71.51 (17)	O5—C15—Nd1^i^	60.4 (4)
O5—Nd1—O5^i^	73.42 (19)	C16—C15—Nd1^i^	170.4 (5)
O2—Nd1—O5^i^	142.16 (17)	C17—C16—C21	120.7 (8)
O11—Nd1—O5^i^	138.86 (17)	C17—C16—C15	118.4 (8)
O6^i^—Nd1—O5^i^	50.26 (16)	C21—C16—C15	121.0 (7)
O3—Nd1—O1	69.33 (18)	C16—C17—C18	120.1 (8)
O4^i^—Nd1—O1	144.00 (17)	C16—C17—H17	120.0
O5—Nd1—O1	137.46 (17)	C18—C17—H17	120.0
O2—Nd1—O1	50.61 (17)	C19—C18—C17	118.3 (9)
O11—Nd1—O1	114.92 (18)	C19—C18—H18	120.8
O6^i^—Nd1—O1	67.75 (17)	C17—C18—H18	120.8
O5^i^—Nd1—O1	106.11 (17)	C20—C19—C18	120.9 (10)
O3—Nd1—Cl1	138.05 (15)	C20—C19—H19	119.5
O4^i^—Nd1—Cl1	79.01 (13)	C18—C19—H19	119.5
O5—Nd1—Cl1	151.08 (13)	C19—C20—C21	120.9 (10)
O2—Nd1—Cl1	86.96 (13)	C19—C20—H20	119.5
O11—Nd1—Cl1	78.90 (13)	C21—C20—H20	119.5
O6^i^—Nd1—Cl1	71.20 (12)	C20—C21—C16	119.0 (8)
O5^i^—Nd1—Cl1	114.46 (12)	C20—C21—H21	120.5
O1—Nd1—Cl1	69.34 (12)	C16—C21—H21	120.5
O3—Nd1—C1	64.6 (2)	O7—C22—O8	119.3 (10)
O4^i^—Nd1—C1	158.9 (2)	O7—C22—C23	121.7 (10)
O5—Nd1—C1	119.2 (2)	O8—C22—C23	118.9 (10)
O2—Nd1—C1	25.4 (2)	O7—C22—Nd2	54.6 (5)
O11—Nd1—C1	96.2 (2)	O8—C22—Nd2	67.1 (5)
O6^i^—Nd1—C1	93.2 (2)	C23—C22—Nd2	165.5 (6)
O5^i^—Nd1—C1	123.6 (2)	C24—C23—C28	121.4 (10)
O1—Nd1—C1	25.83 (19)	C24—C23—C22	121.4 (10)
Cl1—Nd1—C1	80.90 (16)	C28—C23—C22	117.0 (11)
O3—Nd1—C15^i^	80.8 (2)	C23—C24—C25	118.9 (11)
O4^i^—Nd1—C15^i^	75.3 (2)	C23—C24—H24	120.5
O5—Nd1—C15^i^	99.0 (2)	C25—C24—H24	120.5
O2—Nd1—C15^i^	136.5 (2)	C24—C25—C26	118.6 (13)
O11—Nd1—C15^i^	148.63 (19)	C24—C25—H25	120.7
O6^i^—Nd1—C15^i^	24.98 (18)	C26—C25—H25	120.7
O5^i^—Nd1—C15^i^	25.58 (19)	C27—C26—C25	121.2 (13)
O1—Nd1—C15^i^	88.4 (2)	C27—C26—H26	119.4
Cl1—Nd1—C15^i^	91.23 (16)	C25—C26—H26	119.4
C1—Nd1—C15^i^	111.7 (2)	C28—C27—C26	120.4 (14)
O3—Nd1—Nd2	94.43 (15)	C28—C27—H27	119.8
O4^i^—Nd1—Nd2	107.76 (13)	C26—C27—H27	119.8
O5—Nd1—Nd2	160.61 (12)	C27—C28—C23	119.3 (13)
O2—Nd1—Nd2	82.92 (12)	C27—C28—H28	120.4
O11—Nd1—Nd2	120.94 (13)	C23—C28—H28	120.4
O6^i^—Nd1—Nd2	40.66 (12)	O9—C29—O10	125.1 (7)
O5^i^—Nd1—Nd2	90.33 (12)	O9—C29—C30	121.7 (10)
O1—Nd1—Nd2	36.76 (12)	O10—C29—C30	113.1 (9)
Cl1—Nd1—Nd2	46.03 (4)	O9—C29—C30A	118.6 (9)
C1—Nd1—Nd2	61.10 (17)	O10—C29—C30A	116.1 (9)
C15^i^—Nd1—Nd2	65.57 (16)	C30—C29—C30A	9.7 (12)
O8^ii^—Nd2—O10^ii^	79.77 (19)	O11—C36—C37	109.7 (8)
O8^ii^—Nd2—O1	144.98 (19)	O11—C36—H36A	109.7
O10^ii^—Nd2—O1	82.75 (18)	C37—C36—H36A	109.7
O8^ii^—Nd2—O7	127.4 (2)	O11—C36—H36B	109.7
O10^ii^—Nd2—O7	84.2 (2)	C37—C36—H36B	109.7
O1—Nd2—O7	80.1 (2)	H36A—C36—H36B	108.2
O8^ii^—Nd2—O9	74.66 (19)	O12—C37—C36	110.5 (9)
O10^ii^—Nd2—O9	136.64 (18)	O12—C37—H37A	109.6
O1—Nd2—O9	135.61 (19)	C36—C37—H37A	109.6
O7—Nd2—O9	84.3 (2)	O12—C37—H37B	109.6
O8^ii^—Nd2—O6^i^	74.84 (18)	C36—C37—H37B	109.6
O10^ii^—Nd2—O6^i^	73.25 (17)	H37A—C37—H37B	108.1
O1—Nd2—O6^i^	71.14 (17)	O11—C38—C39	111.2 (9)
O7—Nd2—O6^i^	145.2 (2)	O11—C38—H38A	109.4
O9—Nd2—O6^i^	130.15 (18)	C39—C38—H38A	109.4
O8^ii^—Nd2—O8	77.1 (2)	O11—C38—H38B	109.4
O10^ii^—Nd2—O8	66.96 (17)	C39—C38—H38B	109.4
O1—Nd2—O8	122.75 (18)	H38A—C38—H38B	108.0
O7—Nd2—O8	50.7 (2)	O12—C39—C38	111.0 (10)
O9—Nd2—O8	73.49 (17)	O12—C39—H39A	109.4
O6^i^—Nd2—O8	134.43 (17)	C38—C39—H39A	109.4
O8^ii^—Nd2—Cl1	103.71 (14)	O12—C39—H39B	109.4
O10^ii^—Nd2—Cl1	141.60 (14)	C38—C39—H39B	109.4
O1—Nd2—Cl1	72.87 (13)	H39A—C39—H39B	108.0
O7—Nd2—Cl1	119.06 (19)	C10—C9—C14	120.0
O9—Nd2—Cl1	79.17 (13)	C10—C9—C8	120.3 (9)
O6^i^—Nd2—Cl1	71.02 (12)	C14—C9—C8	119.3 (9)
O8—Nd2—Cl1	151.44 (12)	C11—C10—C9	120.0
O8^ii^—Nd2—C22	103.1 (3)	C11—C10—H10	120.0
O10^ii^—Nd2—C22	70.4 (2)	C9—C10—H10	120.0
O1—Nd2—C22	99.2 (2)	C10—C11—C12	120.0
O7—Nd2—C22	25.3 (2)	C10—C11—H11	120.0
O9—Nd2—C22	82.0 (2)	C12—C11—H11	120.0
O6^i^—Nd2—C22	143.3 (2)	C13—C12—C11	120.0
O8—Nd2—C22	26.1 (2)	C13—C12—H12	120.0
Cl1—Nd2—C22	141.6 (2)	C11—C12—H12	120.0
O8^ii^—Nd2—Nd1	111.13 (14)	C14—C13—C12	120.0
O10^ii^—Nd2—Nd1	96.31 (13)	C14—C13—H13	120.0
O1—Nd2—Nd1	41.32 (13)	C12—C13—H13	120.0
O7—Nd2—Nd1	120.26 (16)	C13—C14—C9	120.0
O9—Nd2—Nd1	125.35 (13)	C13—C14—H14	120.0
O6^i^—Nd2—Nd1	40.19 (12)	C9—C14—H14	120.0
O8—Nd2—Nd1	160.40 (12)	C10A—C9A—C14A	120.0
Cl1—Nd2—Nd1	46.23 (4)	C10A—C9A—C8	120.4 (8)
C22—Nd2—Nd1	140.4 (2)	C14A—C9A—C8	119.4 (8)
O8^ii^—Nd2—Nd2^ii^	41.74 (14)	C11A—C10A—C9A	120.0
O10^ii^—Nd2—Nd2^ii^	68.11 (13)	C11A—C10A—H10A	120.0
O1—Nd2—Nd2^ii^	148.79 (13)	C9A—C10A—H10A	120.0
O7—Nd2—Nd2^ii^	85.83 (16)	C10A—C11A—C12A	120.0
O9—Nd2—Nd2^ii^	69.45 (13)	C10A—C11A—H11A	120.0
O6^i^—Nd2—Nd2^ii^	108.84 (12)	C12A—C11A—H11A	120.0
O8—Nd2—Nd2^ii^	35.32 (13)	C13A—C12A—C11A	120.0
Cl1—Nd2—Nd2^ii^	137.78 (5)	C13A—C12A—H12A	120.0
C22—Nd2—Nd2^ii^	61.4 (2)	C11A—C12A—H12A	120.0
Nd1—Nd2—Nd2^ii^	149.02 (2)	C12A—C13A—C14A	120.0
Nd2—Cl1—Nd1	87.74 (5)	C12A—C13A—H13A	120.0
C1—O1—Nd2	153.5 (5)	C14A—C13A—H13A	120.0
C1—O1—Nd1	89.1 (5)	C13A—C14A—C9A	120.0
Nd2—O1—Nd1	101.92 (19)	C13A—C14A—H14A	120.0
C1—O2—Nd1	97.6 (5)	C9A—C14A—H14A	120.0
C8—O3—Nd1	143.1 (6)	C31—C30—C35	120.0
C8—O4—Nd1^i^	134.2 (5)	C31—C30—C29	118.9 (11)
C15—O5—Nd1	159.1 (5)	C35—C30—C29	121.0 (11)
C15—O5—Nd1^i^	94.0 (5)	C32—C31—C30	120.0
Nd1—O5—Nd1^i^	106.58 (19)	C32—C31—H31	120.0
C15—O6—Nd1^i^	95.3 (5)	C30—C31—H31	120.0
C15—O6—Nd2^i^	164.7 (5)	C31—C32—C33	120.0
Nd1^i^—O6—Nd2^i^	99.15 (18)	C31—C32—H32	120.0
C22—O7—Nd2	100.2 (6)	C33—C32—H32	120.0
C22—O8—Nd2^ii^	169.6 (7)	C32—C33—C34	120.0
C22—O8—Nd2	86.8 (6)	C32—C33—H33	120.0
Nd2^ii^—O8—Nd2	102.9 (2)	C34—C33—H33	120.0
C29—O9—Nd2	135.2 (5)	C33—C34—C35	120.0
C29—O10—Nd2^ii^	139.3 (5)	C33—C34—H34	120.0
C36—O11—C38	109.3 (7)	C35—C34—H34	120.0
C36—O11—Nd1	128.6 (5)	C34—C35—C30	120.0
C38—O11—Nd1	122.1 (5)	C34—C35—H35	120.0
C39—O12—C37	105.8 (8)	C30—C35—H35	120.0
O2—C1—O1	119.9 (8)	C31A—C30A—C35A	120.0
O2—C1—C2	120.4 (8)	C31A—C30A—C29	122.2 (10)
O1—C1—C2	119.5 (8)	C35A—C30A—C29	117.7 (10)
O2—C1—Nd1	57.0 (4)	C32A—C31A—C30A	120.0
O1—C1—Nd1	65.1 (4)	C32A—C31A—H31A	120.0
C2—C1—Nd1	160.5 (5)	C30A—C31A—H31A	120.0
C7—C2—C3	119.5 (8)	C31A—C32A—C33A	120.0
C7—C2—C1	118.6 (8)	C31A—C32A—H32A	120.0
C3—C2—C1	121.8 (8)	C33A—C32A—H32A	120.0
C4—C3—C2	119.6 (9)	C34A—C33A—C32A	120.0
C4—C3—H3	120.2	C34A—C33A—H33A	120.0
C2—C3—H3	120.2	C32A—C33A—H33A	120.0
C5—C4—C3	120.4 (9)	C33A—C34A—C35A	120.0
C5—C4—H4	119.8	C33A—C34A—H34A	120.0
C3—C4—H4	119.8	C35A—C34A—H34A	120.0
C4—C5—C6	120.0 (10)	C34A—C35A—C30A	120.0
C4—C5—H5	120.0	C34A—C35A—H35A	120.0
C6—C5—H5	120.0	C30A—C35A—H35A	120.0

Symmetry codes: (i) −*x*+1, −*y*, −*z*+1; (ii) −*x*+1, −*y*+1, −*z*+1.
